# No component loosening of a cementless deep dish rotating platform knee at a 5-year follow-up

**DOI:** 10.1007/s00167-022-07113-0

**Published:** 2022-08-15

**Authors:** Christian Stadler, M. Hofstätter, M. Luger, M. Stöbich, B. Ruhs, T. Gotterbarm, A. Klasan

**Affiliations:** 1 Johannes Kepler University Linz, Altenberger Str. 96, 4040 Linz, Austria; 2 Department for Orthopedics and Traumatology, Kepler University Hospital GmbH, Krankenhausstr. 9, 4020 Linz, Austria; 3 Orthopedics and Traumatology, Klinik Diakonissen, Weißenwolffstr. 13, 4020 Linz, Austria; 4 Orthopedics, Klinik Diakonissen, Weißenwolffstr. 13, 4020 Linz, Austria; 5 AUVA Trauma Hospital Styria Graz, Göstinger Str. 24, 8020 Graz, Austria

**Keywords:** Cementless TKA, Vanguard cementless, Rotating platform knee, Aseptic loosening, Radiolucent lines

## Abstract

**Purpose:**

Cemented fixation remains the gold standard in total knee arthroplasty. With an increasing number of younger patients undergoing total knee arthroplasty and a growing patient population demanding higher physical activity, a rising interest in discussion of cementless fixation is notable. The current scientific literature does not give a clear recommendation for or against uncemented total knee arthroplasty. The purpose of this study was the investigation of the 5-year clinical and radiographic outcomes of a cementless deep-dish rotating platform implant.

**Methods:**

A total of 91 primary cementless total knee arthroplasties were included in this single-centre prospective observational study. The primary outcome was revision rate due to aseptic component loosening. Further outcome measures were assessment of the of the radiographic outcome as well as the clinical outcome based on Range of Motion and scores such as American Knee Society Score, Oxford Knee Score, Knee Injury and Osteoarthritis Outcome Score and European Quality of Life 5 Dimension 3 Level at a follow-up of 5 years.

**Results:**

Mean age of the study population was 67.3 ± 6.6 years with 49.5% of the participants being female. Aseptic component loosening occurred in none of the patients. Implant survival with revision for any reason as endpoint was 97.8% (95% CI 100–96%) and 95.6% (95% CI 100–94%) with reoperation of any cause as endpoint. Radiolucent lines were detected in a total of eight cases (8.8%) and disappeared within the first year after surgery in five cases. Total Range of Motion improved significantly from 106° ± 15° preoperatively to 118° ± 10° at final FU (*p* < 0.001). All investigated scores improved significantly after total knee arthroplasty.

**Conclusion:**

The results of this study reveal excellent mid-term performance of a cementless deep dish rotating platform total knee implant, with no component loosening, very low overall revision rate, only temporarily present radiolucent lines in a minority of patients and excellent clinical results. Therefore, cementless total knee arthroplasty is an appropriate treatment option for patients with severe osteoarthritis of the knee.

**Level of evidence:**

Level II (prospective cohort study).

## Introduction

Cemented fixation remains the gold standard in TKA, demonstrating successful outcomes with low rates of aseptic loosening in long-term follow-up (FU) [13, 21].

In recent years, cementless fixation has gained more interest for several different reasons. The theoretical advantages of cementless fixation in TKA include the potential preservation of native bone stock, the avoidance of cement debris and, especially, the potential of achieving a long-lasting and biological fixation [8]. As the number of younger patients undergoing TKA is constantly increasing, there is also an increasing number of patients demanding higher physical activity resulting in mechanical stress on the implant with higher probability of possible secondary surgery [8, 15, 34]. Therefore, a rising interest in discussion of cementless fixation is notable. In studies comparing both fixation techniques, uncemented TKA shows comparable short-term and mid-term outcomes without any statistical advantage for one method over the other [9, 22, 37]. Hence, the current scientific literature does not give a clear recommendation for or against uncemented TKA.

The purpose of this study was the investigation of the 5-year clinical and radiologic outcome in patients who underwent TKA with the cementless Vanguard Deep Dish Rotating Platform Knee (Zimmer Biomet, Warsaw, Indiana, U.S.). The hypothesis of this study was that cementless rotating platform TKA is an appropriate and safe treatment option for patients with severe osteoarthritis (OA) of the knee with low revision rates and good clinical outcomes. This is the first prospective study investigating the mid-term results of the specific implant mentioned above.

## Materials and methods

### Study population

This is a single-arm, single-centre, prospective, observational study of performance and safety of the uncemented Vanguard Deep Dish Rotating Platform Knee (Zimmer Biomet, Warsaw, Indiana, U.S.). The performance was assessed by the rate of aseptic loosening within 5 years of FU (primary outcome measure), radiographic evaluations as well as various clinical scores (secondary outcome measure). Consecutive patients who have undergone primary TKA using the implant mentioned above between 2013 and 2016 with a FU of 5 years were included in this study. Indications for performing TKA were painful and disabled joints caused by primary OA, posttraumatic OA and rheumatoid OA that had failed conservative treatment. Further inclusion criteria were ability and willingness to follow instructions regarding the postoperative treatment and rehabilitation, returning to FU evaluations as well as full skeletal maturity (minimum age 18 years) and a signed and understood consent form. Exclusion criteria were valgus and varus deformities > 15° or insufficient collateral ligaments requiring increased constraint, infection, osteomyelitis, previous partial or total knee replacement, previous osteotomy, skeletal immaturity as well as relative contraindications including uncooperative patients, patients with neurological disorders and incapability of following therapeutical instructions, severe osteoporosis, metabolic disorders, which may impair bone formation, osteomalacia, distant foci of infections, vascular inefficiency, neuromuscular diseases and incomplete or deficit soft tissue surrounding the knee.

### Implant and surgical procedure

#### Implant

The cementless Vanguard Deep Dish Rotating Platform Knee (Zimmer Biomet, Warsaw, Indiana, U.S.) consists of three main primary components: Femoral, tibial and a bearing component, with the option of patella resurfacing. It uses the standard Vanguard Cruciate Retaining femoral component, which is anatomically shaped and made from cobalt–chromium molybdenum alloy with a porous coated fixation surface and a highly polished articulating surface. The finned tibial component is made of cobalt–chromium molybdenum alloy with a blasted fixation surface having a porous and hydroxyapatite coating and a highly polished articulating surface. The cruciate retaining bearing component is manufactured from ArCom^®^ Ultra High Molecular Weight Polyethylene (UHMWPE) with a compression molded tibiofemoral articulation and a machined bearing interface. Coronally, it is matched to the curved shape of the femoral component while sagittal, it has a single radius in accordance with the femoral component’s shape in extension. Inferiorly, the surface is shaped planarly with a pivot post, which latches in the tapered hole of the tibial components and allows for rotation of the bearing component.

#### Surgical procedure

A single shot of 1.5 g cefuroxime or 600 mg clindamycin in case of penicillin allergy was administered as perioperative prophylaxis. There were mainly three participating orthopaedic surgeons who performed surgery according to the manufacturer’s instructions. Tourniquet was used in all cases and bone surfaces were tap-dried prior to component implantation. If necessary, lateral retinacular release was performed to ensure centralized patella tracking. Full weight bearing was permitted to all patients immediately, although usage of two crutches was recommended to each patient for 6 weeks after surgery. Thromboembolic prophylaxis was prescribed to every patient postoperatively (Low molecular weight heparin in prophylactic dosage for 4 days followed by 10 mg Rivaroxaban for 10 days). Physiotherapy was provided to each patient once per day within the hospital. Further outpatient physiotherapy was prescribed after discharge from hospital.

No experimental or investigational devices were used during this study.

### Follow-up

#### Data collection

Data collection was conducted preoperatively, intraoperatively and postoperatively at the study centre as well as within FU visits at the outpatient clinic after 3 months, 1 year, 2 years, 3 years (optionally by phone) and 5 years. Each FU visit time point was determined based on surgery date with a maximum time window of 3 months, which was also necessary due to the ongoing COVID-19 pandemic.

#### Implant survival and adverse events

Reoperations of any cause as well as adverse events were registered throughout the whole FU and were categorized according to the definitions according to the standardized list and definitions of the Knee Society published by Healy et al. [12].

#### Radiographic assessment

Radiographic assessments consisted of weight bearing anterior posterior, skyline at 30° flexion and mediolateral view to evaluate integrity, positioning and radiolucent lines (RLL) according to the evaluation system described by Meneghini et al. [19]. As for the evaluation of the implant positioning, coronal femoral (*α*) and tibial component angle (*β*) were measured as medial angle between the anatomical femoral shaft axis and a connecting line between the medial and lateral condyle of the femoral component (*α*) as well as medial angle between the anatomical tibial axis and the axis of the fixation surface of the tibial component (*β*). Sagittal femoral (*γ*) and tibial component angle (*σ*) were measured as the angle between the anatomical femoral axis and the line perpendicular to the distal fixation surface containing the pegs of the femoral component (*γ*) as well as the angle between the anatomical tibial axis and the axis of the fixation surface of the tibial component (*σ*) according to the previously published technique described by Meneghini et al. [19]. The radiographic assessment was conducted by two authors independently using IMPAX (EE R20 XVI; Agfa HealthCare, Mortsel, Belgium).

#### Range of motion and patient reported outcome measures

Data to determine the clinical and functional performance were collected pre- and postoperatively applying the “American Knee Society Score” (AKSS), “Oxford Knee Score” (OKS), “Knee Injury and Osteoarthritis Outcome Score” (KOOS) and “European Quality of Life 5 Dimension 3 Level” (EQ5D). Range of Motion (ROM) was measured using a long arm goniometer [10].

This study was approved prior to commencement by the ethical review committee of the Medical Faculty of the Johannes Kepler University Linz (former ethical review committee of Upper Austria) and registered as “B-53–13” and “BMETEU.CR.EU 13”.

### Statistical analysis

SPSS (version 26.0, IBM, Armonk, NY) was used for the statistical analysis. Kolmogorov–Smirnov test was performed to test for normal distribution. As for metric scaled data, arithmetic mean value and the standard deviation were calculated. These two parameters were reported as mean ± standard deviation. As for non-normally distributed metric scaled parameters, Wilcoxon Signed Rank test was performed to analyze the significance of the difference between those parameters, while *t* test was performed if the metric scaled parameters were normally distributed. Kaplan–Meier survival analysis was performed to analyze the survival rate of the TKA. Patients lost to FU for any reason were censored at the respective FU, if no adverse events were documented. Intra-rater and inter-rater agreements were calculated using interclass correlation coefficient (ICC). Blinded repeated measurements for intra-rater reliability were conducted at a time interval of 1 week. A sample size of 90 was calculated using data regarding the incidence of aseptic loosening available at the time the study was planned to detect the primary outcome [1]. The level of significance was defined at *p* ≤ 0.05.

## Results

### Study population and surgical information

**Fig. 1:**
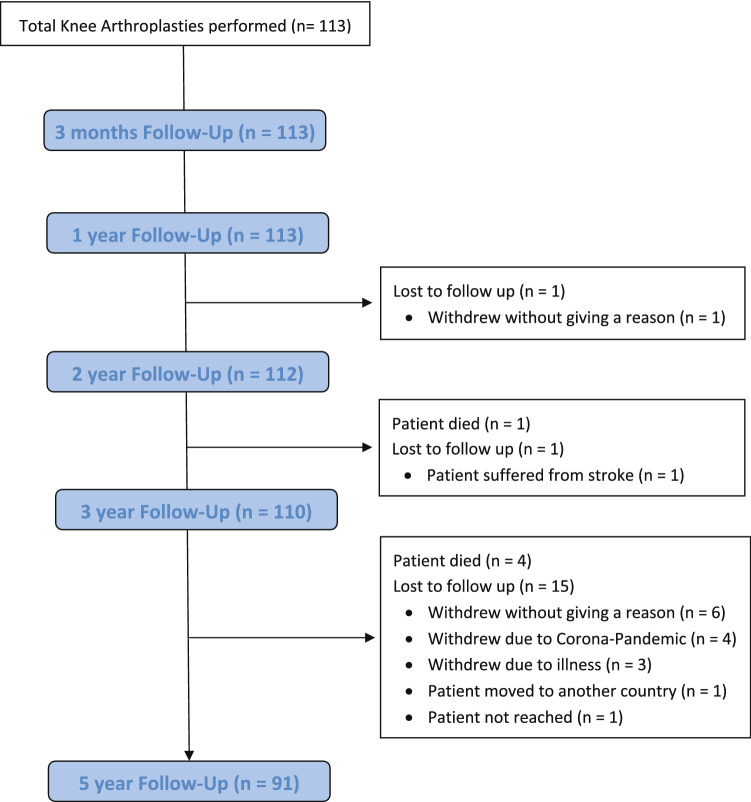
Overview of the follow-up and the patients lost to follow-up

At final FU, 91 patients were available with an average FU of 5.0 ± 0.1 years (Fig. 1, Table 1). Any adverse events in patients that did not reach the 5-year FU were accounted for.

**Table 1:** Baseline characteristics of the study population including average implant sizes and indications for TKA

Demographic characteristics	Overall (*n* = 91)
Mean follow-up (years)	5.0 ± 0.1
Mean age (years)	67.3 ± 6.6
Female	45 (49.5%)
Male	46 (50.5%)
Right Knee	52 (57.1%)
Left Knee	39 (42.9%)
Mean body weight (kg)	91 ± 16
Mean body height (cm)	171 ± 9
Mean BMI	31 ± 5
Mean femoral component	70 ± 5
Mean size tibial component	5 ± 1
Mean size polyethylene inlay	10 ± 1
Indications for TKA	
Primary Osteoarthritis	86 (94.5%)
Posttraumatic Osteoarthritis	5 (5.5%)

A mid-vastus approach was performed in 77 cases (84.6%) and a medial parapatellar approach in 14 cases (15.4%). Lateral retinacular release was performed in 24 cases (26.4%). Patella resurfacing was not performed.

### Implant survival and adverse events

**Fig. 2:**
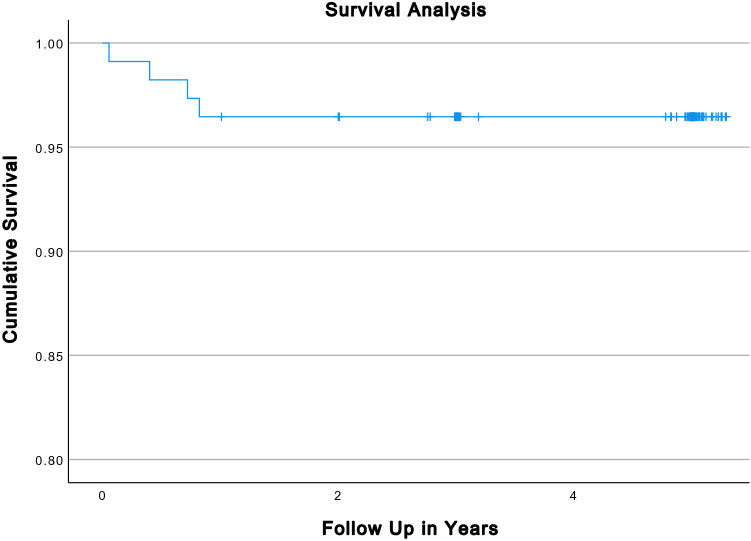
Kaplan–Meier survival analysis with reoperation of any cause as endpoint

Within the FU, there were two revisions and two reoperations as mentioned below. This resulted in an implant survival rate of 97.8% (95% CI 100–96%) with revision as endpoint (2 out of 91;) and 95.6% (95% CI 100–94%) with reoperation of any cause as endpoint (4 out of 91; Fig. 2) at the 5 years FU. No aseptic loosening was observed within the FU. Accordingly, implant survival rate with aseptic loosening as endpoint was 100% (0 out of 91).

A total of six device related adverse events occurred within the FU period. There was one case of an early periprosthetic infection occurring 3 weeks after initial surgery, which was managed with a DAIR (Fucidin acid for 6 weeks for *staphylococcus epidermidis*). In one case, lateralization of the patella with lateral patellar compression syndrome including pain was resolved by performing a lateral release and a patella resurfacing 10 months after the initial surgery. One patient sustained a periprosthetic distal femoral fracture 5 months after the initial surgery. Intraoperatively, the femoral component was well fixed and the fracture was successfully managed with primary fixation. In one case, postoperative wound dehiscence occurred and was managed with superficial wound revision. Another patient fell on the operated knee and suffered a bony avulsion of the medial collateral ligament which was treated conservatively using a brace. There was one case of arthrofibrosis, which was successfully treated by mobilization of the knee under anaesthesia at the 10-week mark.

### Radiographic assessment

Radiographic assessment of the implant revealed no significant change in implant positioning within the FU (*p* > 0.05; Table 2).

**Table 2:** Results of the radiographic analysis evaluating the implant position within the follow-up

	Immed. postop.	3 mo. postop.	1 y. postop.	2 y. postop.	5 y. postop.
α	95.6° ± 1.0°	95.4° ± 1.2°	95.5° ± 1.3°	95.2° ± 1.6°	95.2° ± 1.5°
β	90.3° ± 1.2°	90.0° ± 1.3°	90.0° ± 1.7°	90.0° ± 1.3°	90.2° ± 1.5°
γ	1.3° ± 1.3°	1.3° ± 1.0°	1.4° ± 1.1°	1.4° ± 1.0°	1.4° ± 1.1°
σ	85.5° ± 1.6°	85.4° ± 1.6°	85.6° ± 1.7°	85.6° ± 2.0°	85.6° ± 2.4°

**Fig. 3:**
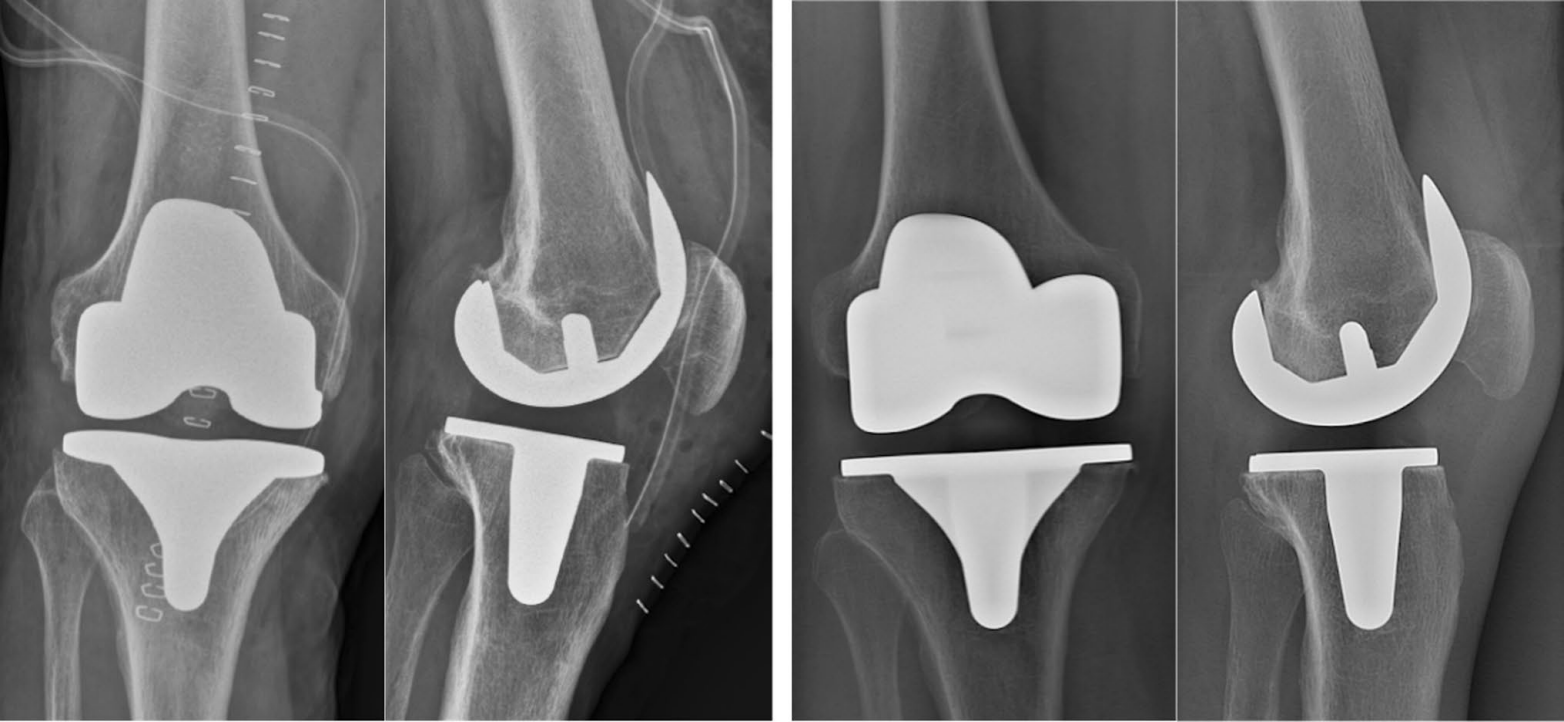
Temporary postoperative radiolucent lines in the lateral view. The X-rays on the left show the immediate postoperative result with a radiolucent line in Zone 3A at the lateral view with no residual radiolucent line in that zone at the X-ray 3 months after TKA in the same patient

RLL surrounding the femoral or tibial component in one or more zones were detected in a total of eight cases (8.8%; Table 3, Fig. 3). All detected radiolucent lines had a width of 1 mm except for one case where the radiolucent line at zone 3 of the femoral component measured 4 mm only at the immediate postoperative radiograph.

**Table 3:** Results of the radiographic analysis regarding the presence of radiolucent lines within the follow-up

Zone	Immed. postop.	3 mo. postop.	1 y. postop.	2 y. postop.	5 y. postop.
Zone 1 Femoral	–	–	1 (1.1%)	–	–
Zone 2 Femoral	–	–	–	1 (1.1%)	1 (1.1%)
Zone 3 Femoral	2 (2.2%)	1 (1.1%)	1 (1.1%)	1 (1.1%)	1 (1.1%)
Zone 3A Femoral	4 (4.4%)	1 (1.1%)	2 (2.2%)	1 (1.1%)	1 (1.1%)
Zone 3P Femoral	–	–	–	1 (1.1%)	1 (1.1%)
Zone 5 Femoral	–	–	–	–	–
Zone 1 Tibial a.p.	–	–	–	–	–
Zone 2 Tibial a.p.	–	–	–	–	–
Zone 3M Tibial a.p.	–	–	–	–	–
Zone 3L Tibial a.p.	–	–	–	–	–
Zone 5 Tibial a.p.	–	–	–	–	–
Zone 1 Tibial lat.	–	–	–	–	–
Zone 2 Tibial lat.	–	–	–	–	–
Zone 3A Tibial lat.	1 (1.1%)	–	–	–	–
Zone 3P Tibial lat.	–	–	–	–	–
Zone 5 Tibial lat.	–	–	–	–	–

Intra-rater ICC was 0.93, while inter-rater ICC was 0.89.

### Range of motion

Compared to the values collected preoperatively, all postoperatively collected values regarding the ROM (Extension, Flexion and overall ROM) improved significantly (*p* < 0.05; Table 4).

**Table 4:** Comparison of the preoperative ROM and the ROM at final follow-up

	Preop.	5 y. postop.	*p *value
Extension	− 3° ± 5°	0° ± 1°	< 0.001
Flexion	109° ± 13°	118° ± 9°	< 0.001
ROM	106° ± 15°	118° ± 10°	< 0.001

### Patient reported outcome measures (AKSS, OKS, KOOS, EQ5D)

**Fig. 4:**
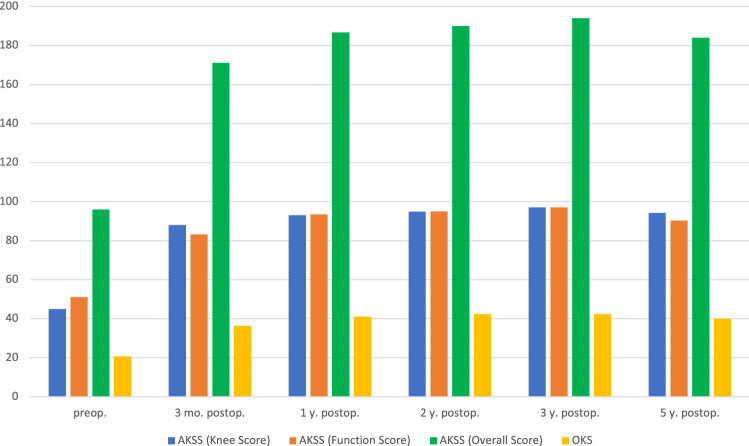
Overview of the AKSS and OKS within the follow-up

The AKSS and the OKS showed a significant improvement at every FU when compared to the preoperative initial score (*p* < 0.05; Fig. 4). The KOOS and the EQ5D improved significantly in every subsection within the FU as well (*p* < 0.05; Table 5).

**Table 5:** Overview of the KOSS and EQ5D within the follow-up

	Preop.	3 mo. postop.	1 y. postop.	2 y. postop.	3 y. postop.	5 y. postop.
KOOS symptoms	44%	76%	86%	86%	89%	87%
KOOS pain	35%	80%	89%	90%	92%	88%
KOOS activities of daily living	40%	83%	90%	90%	92%	88%
KOOS sport/recreation	7%	49%	66%	70%	72%	68%
KOOS quality of life	27%	70%	80%	81%	82%	78%
KOOS overall score	35%	76%	85%	86%	88%	85%
EQ5D mobility	2.0	1.3	1.2	1.1	1.1	1.1
EQ5D selfcare	1.9	1.1	1.0	1.0	1.0	1.0
EQ5D activity	2.0	1.2	1.1	1.1	1.0	1.1
EQ5D pain	2.3	1.6	1.3	1.2	1.2	1.4
EQ5D anxiety	1.4	1.1	1.1	1.1	1.1	1.1
EQ5D health state	52.4	81.1	87.6	90.1	90.4	83.0

## Discussion

The most important findings of the present study are excellent mid-term outcomes—including implant survival as well as radiographic and clinical outcomes—of the cementless Vanguard Deep Dish Rotating Platform Knee. This is the first prospective study reporting mid-term outcomes of the specific implant mentioned above.

There was a relatively low overall revision rate of 2.2% at 5 years FU with no observed case of aseptic component loosening. This is in line with other recent reports, also reporting no loosening of the uncemented tibial or femoral component after cementless TKA [30, 31]. Other reports revealed promising results regarding the revision rate due to aseptic loosening after cementless TKA with just slightly higher revision rates as well [20, 25]. Some authors, however, reported higher revision rates due to aseptic loosening after cementless TKA [3, 5]. As for possible differences regarding the rates of aseptic loosening between mobile and fixed bearing TKA previous studies revealed no significant differences between those two implant designs [11]. Nevertheless, there are hardly recent reports comparing exclusively cementless mobile bearing and fixed bearing implant designs regarding possible differences in aseptic loosening rates.

The implant survival rate within this study of 97.8% with revision as endpoint is matching results from a systematic review of the literature [18]. Comparing survival rates of cementless and cemented TKA in general, there are reports of better all-cause survivorship of cementless fixation in TKA [23], while there are also reports stating no difference in mid-term survival between cementless and cemented fixation [25, 37]. Regarding specific patient populations, cementless TKA showed a significantly lower revision rate in morbidly obese patients [2] and superior clinical outcomes with equal implant survival compared to cemented TKA in patients younger than 65 years [35].

As for the implant related complications, one case (0.9%) of periprosthetic infection occurred, which is less compared to data reported in literature [32]. Insufficient ROM leading to manipulation under anaesthesia to improve ROM was evident in one patient postoperatively (1.1%). This is also slightly less compared to other reports according to a review of literature conducted by Kornuijt et al. [14], who found rates of manipulations under anaesthesia ranging from 1.3 to 13.5% after TKA.

The radiographic evaluation revealed no significant changes in implant position within the FU (Table 3), which is in contrast to cementless unicondylar knee arthroplasty, where changes in the posterior tibial slope over time are reported [33].

Within this study, RLL, a major concern for many surgeons due to previous reports [7], occurred and disappeared within the first year after surgery. Only in one case, RLL persisted throughout the study period, but without any clinical signs of component loosening. This phenomenon has been previously described by other authors and is possibly caused by imprecise bone cuts or component positioning [36]. Regarding the difference between the occurrences of RLL in cementless vs. cemented TKA, there are various reports with different outcomes. Some authors report less RLL in cementless TKA [6], while some authors report more RLL in cementless TKA [9] and others report no difference between cementless and cemented TKA [37]. With aseptic loosening still being one of the most frequent indications for revision in TKA [28], there is a special interest in the occurrence and change over time of RLL as they also seem to be associated with dissatisfaction and pain after TKA [27]. However, the reasons for and factors leading to RLL in TKA are still subject of discussion in recent literature as they seem to be less dependent on implant design and might be triggered by implant positioning or surgical technique [4, 29]. Another reason for early RLL is bone remodeling during the ingrowth phase, during which stress shielding might occur [36].

The ROM improved at a 5-year FU (Table 4), which matches the reports of other authors in recent literature [5, 35]. This allows for managing most tasks of everyday life and is well above the minimum flexion limit of 90°, which is needed to adequately walk a set of stairs [16].

Additionally, all evaluated clinical and patient reported outcome scores including AKSS, OKS, KOOS and EQ5D showed significant improvements within the FU when compared to the preoperatively assessed values. Similar results were retrieved by other authors, who also reported a significant improvement of PROMS after cementless TKA [26].

Despite the prospective study design, there are some limitations to this study that must be considered. The patient selection for this study was conducted by the surgeons who performed TKA. Therefore, there might be a selection bias, as some patients (for example with severe osteoporosis or severe obesity) might have more likely been treated with a cemented implant alternative that was available at the centre during the study period. There were, however, no intraoperative changes of fixation due to potential bone density issue. Three surgeons performed the surgeries, which is reflected in different surgical approaches. We do not expect that the differences in approach would affect the mid-term results [17]. The surgeons were experienced in TKA. Therefore, the results of this study are not necessarily applicable on surgeons with less experience in TKA. The study was designed as a single-arm prospective study, without a comparative group. Although TKA using other implants was performed at the study centre during the study period, it was decided to solely investigate the implant mentioned above as the other implant used at the study centre was cemented with a fixed bearing inlay, which are two important and distinct variables that might have had a significant impact on the primary outcome measure. The implant can nowadays be considered of an older design, since the manufacturer now offers a more modern implant albeit not a cementless version as of writing of the manuscript. Similarly, mobile bearing is also being less used in primary arthroplasty [24]. Nevertheless, this design demonstrates encouraging results even in this version. Although component loosening was not observed, RLL were detected within the FU. However, potential component migration using radiostereometric analysis was not performed. Similarly, bone mineral density around the implant was not assessed. Lastly, the ongoing COVID-19 pandemic complicated the FU significantly. A time window of 3 months based on surgery date was allowed for FU visits, which resulted in a few patients being investigated slightly before full 5 years after surgery elapsed. Additionally, strict hygienic precautions and other pandemic related factors discouraged patients from completing the 5-year FU and, therefore, resulted in a higher number of patients lost to FU.

The present study as well as the majority of the literature demonstrate at least comparable outcomes of cementless TKA and cemented TKA. Surgeons might want to consider cementless TKA in their practice and manufacturers might want to expand their portfolio by including a cementless version of their modern implants.

## Conclusion

The results of this study reveal excellent mid-term performance of a cementless deep dish rotating platform implant, with no aseptic loosening, very low revision rate, only temporarily present RLL in a minority of patients and excellent clinical results. Therefore, cementless TKA is an appropriate treatment option for patients with severe OA of the knee.
